# Depersonalization and depressive symptoms in two online samples: development and initial validation of a short Depersonalization Mechanism Scale

**DOI:** 10.3389/fpsyt.2026.1892456

**Published:** 2026-08-05

**Authors:** Dominika Fortuna, Krystyna Golonka

**Affiliations:** 1 Doctoral School in the Social Sciences, Jagiellonian University, Krakow, Poland; 2 Department of Crisis Intervention and Psychotherapy, Institute of Applied Psychology, Faculty of Management and Social Communication, Jagiellonian University, Krakow, Poland

**Keywords:** depersonalization, depression, emotion regulation, psychometrics, surveys and questionnaires

## Abstract

**Background:**

Depersonalization can be conceptualized as an emotion regulation mechanism that may emerge in response to prolonged stress and overstimulation. Individuals differ in their tendency to rely on this mechanism, with varying levels of depersonalization experiences observed in the general population. The present study aimed to develop and validate a brief version of Depersonalization Mechanism Scale. Scale reduction was undertaken to improve measurement efficiency and reduce respondent burden while preserving adequate coverage of the underlying construct. Additionally, associations between depersonalization and depressive symptoms were examined.

**Methods:**

Two independent samples from the general Polish population participated in the study. Study 1 included 204 adults aged 22–86 years, whereas Study 2 included 367 adults aged 19–30 years. Following an initial item reduction procedure informed by psychometric and content-related considerations, the factor structure of the shortened DMS was examined using exploratory factor analysis in Study 1 and confirmatory factor analysis in Study 2. Participants completed a brief version of Depersonalization Mechanism Scale (DMS), Patient Health Questionnaire (PHQ-9), and Occupational Depression Inventory (ODI).

**Results:**

The final version of the DMS comprised 14 items grouped into two subscales: Detachment and Emotional Numbness. The abbreviated scale demonstrated high internal consistency and retained the theoretical structure of the original measure. Confirmatory factor analysis supported both a two-factor and a bifactor representation of the scale. Correlation and regression analysis indicated that the depersonalization mechanism is significantly associated with depressive symptoms.

**Conclusions:**

The findings support the reliability and validity of the shortened DMS for examining depersonalization as an emotion regulation mechanism. Depersonalization was strongly associated with depressive symptoms in a non-clinical sample, highlighting its relevance to emotional functioning. The brief format of the 14-item DMS may facilitate future research on depersonalization in large-scale and multivariate studies.

## Introduction

The clinical presentation of depersonalization is characterized by recurrent or prolonged episodes during which individuals experience themselves as unreal, detached from their own person, or as if they were observing their thoughts, emotions, sensations, or behaviors from outside themselves (1) Simeon (2) identified two core components of depersonalization disorder: detachment and hypoemotionality (emotional numbing or blunting). According to DSM-5, depersonalization frequently co-occurs with unipolar depressive disorder, a relationship consistently observed in clinical populations (1). However, considerably less is known about the role of depersonalization in non-clinical samples. In our previous work, we introduced a method for assessing depersonalization symptoms in a non-clinical population and demonstrated associations with cognitive and behavioral emotion regulation strategies (3). The present study extends this line of research by further operationalizing depersonalization and examining its role within a broader framework of emotion regulation.

Emotion regulation refers to the processes through which individuals influence the occurrence, intensity, duration, and expression of emotions (4). Effective regulation is essential for adaptive functioning and psychological well-being, whereas disturbances in emotion regulation have been linked to a broad range of psychological difficulties, including depression, dissociative symptoms, and post-traumatic stress disorder (5–8). Although emotion regulation research has traditionally focused on consciously deployed cognitive or behavioral strategies (9, 10), growing attention has been directed toward more automatic regulatory mechanisms that operate with limited conscious control (7). Distinguishing between these levels of regulation may be particularly important for understanding psychological responses that emerge spontaneously under conditions of emotional overload or stress.

Within this framework, depersonalization may be conceptualized as an emotion regulation mechanism rather than an emotion regulation strategy. Unlike deliberate regulatory actions, depersonalization is generally not intentionally initiated by the individual. Instead, it tends to emerge automatically in response to overwhelming stress, trauma, or excessive psychological demands (11, 12). By creating a sense of detachment from emotional experience, depersonalization may temporarily reduce emotional intensity and facilitate functioning under adverse circumstances (13, 14). Although such detachment may serve an adaptive regulatory function in the short term (3, 15), persistent reliance on depersonalization may become maladaptive and contribute to impaired interpersonal functioning and broader psychological distress (16). Longitudinal evidence indicates that depersonalization predicts subsequent post-traumatic stress symptoms among frontline healthcare workers exposed to pandemic-related stressors (17). Similarly, Michal et al. (18) demonstrated that depressive patients presenting depersonalization symptoms were more likely to experience recurrence or persistence of depression over a five-year follow-up period. These findings suggest that depersonalization may initially function as a protective mechanism but, when chronically activated, may contribute to adverse mental health outcomes. However, the regulatory processes underlying depersonalization remain insufficiently understood.

Consistent with this perspective, our previous findings showed that depersonalization was associated not only with maladaptive strategies, such as self-blame, blaming others, rumination, catastrophizing, withdrawal, and ignoring, but also with strategies commonly regarded as adaptive, including acceptance, positive refocusing, positive reappraisal, putting events into perspective, seeking distraction, and seeking social support (3). The coexistence of this associations suggests that depersonalization may represent a multifaceted regulatory mechanism that interacts with a broad range of regulatory processes. These findings highlight the need for further investigation of its underlying structure and psychological correlates.

Despite increasing interest in depersonalization, significant gaps remain in the literature. First, relatively little is known about the latent structure of depersonalization conceptualized as an emotion regulation mechanism, particularly in non-clinical populations. Second, although depersonalization has repeatedly been linked to depressive symptoms, it remains unclear whether its specific components differentially contribute to depression-related outcomes. Addressing these questions requires psychometrically validated instruments capable of efficiently assessing depersonalization mechanisms in studies examining multiple psychological constructs simultaneously.

In our previous work, we developed the Depersonalization Mechanism Scale (DMS) to assess depersonalization as an emotion regulation mechanism in non-clinical populations (3). However, the length of the original instrument may limit its applicability in large-scale and multivariate studies. Developing a shorter version of the DMS may facilitate broader investigation of depersonalization mechanisms while preserving the theoretical structure of the construct and maintaining satisfactory psychometric properties. Such instruments are particularly valuable in research seeking to integrate depersonalization into broader models of emotion regulation and psychological vulnerability.

The present study had two complementary objectives. First, we developed and validated a shortened version of the Depersonalization Mechanism Scale (DMS), examining its factor structure and reliability in a non-clinical sample. Second, using the validated short form, we investigated the associations between depersonalization components and depressive symptoms. By integrating psychometric validation with the examination of psychological correlates, the present study aims to advance understanding of depersonalization as an emotion regulation mechanism and clarify its relevance to depressive symptomatology in non-clinical populations.

## Materials and methods

### Participants

Two independent samples of Polish adults participated in the study. Because the primary aim of Study 1 was to examine the relationship between depersonalization and depressive symptoms in a broad adult population, a wide age range was used. In contrast, Study 2 focused specifically on young adults to examine this relationship within a more homogeneous group.

Study 1 included 204 Polish participants aged between 22 and 86 years (M_age_=41.8, SD, 14.4). Participants were recruited in 2023 by the authors and the Polish national research panel Biostat. The sample consisted of 119 women (58.3%) and 85 men (41.7%). For Study 1, the inclusion criteria were being at least 18 years of age and being currently employed. No upper age limit was imposed, and no additional exclusion criteria were applied. Participants recruited through the Biostat research panel were selected using the panel’s profiling system. According to the panel provider, participant information is regularly verified, multiple account creation is not permitted, and response quality is monitored to identify inattentive or random responding. Survey access is controlled through individual participant credentials. Only fully completed questionnaires were included in the analyses.

Study 2 included 367 participants aged between 19 and 30 years (M_age_=25.9, SD, 3.21). Participants were recruited in 2024 through the Polish national research panel Ariadna. The sample comprised 181 men (49.3%), 184 women (50.1%), and two participants (0.5%) who did not identify as either a man or a woman. For Study 2, the sole inclusion criterion was being between 18 and 30 years of age; no exclusion criteria were applied. According to the panel provider, panelists undergo verification procedures designed to ensure that registered accounts correspond to real individuals and to prevent multiple registrations by the same person. The panel provider also reports that its quality assurance procedures are subject to independent audits within the framework of the Polish Interviewer Quality Control Program (PKJPA). Only fully completed questionnaires were included in the analyses.

Participation in both studies was voluntary, and informed consent was obtained prior to participation. The study was conducted with accordance of the principles of Declaration of Helsinki and American Psychological Association’s Ethical Principles of Psychologists and Code of Conduct. According to applicable national regulations and institutional guidelines, ethical review and approval were not required for this study as it involved an anonymous, non-interventional survey with no collection of identifiable personal data.

### Design and procedure

An item reduction procedure was undertaken to develop a more concise yet psychometrically robust measure. The development of a shorter version was motivated by both practical and methodological considerations. Lengthy studies may discourage participation (19, 20), making it more difficult to recruit the required sample efficiently. Moreover, long self-report instruments often contain repetitive items assessing similar content, which can increase respondent burden, contribute to fatigue and disengagement, and ultimately reduce response quality (21). Consequently, brief instruments are particularly advantageous in online settings, where participant attention and retention are crucial.

Item retention decisions were informed by both psychometric criteria and theoretical considerations derived from content analysis. Items exhibiting substantial conceptual overlap or redundancy were removed to improve measurement efficiency without compromising psychometric quality or construct coverage. This process reduced the original DMS from 41 to 18 items, grouped into two subscales: Detachment and Emotional Numbness. The initial item reduction procedure yielded an 18-item version of the DMS, which was subsequently subjected to further refinement.

To evaluate whether the factor structure of the original DMS would be retained in the abbreviated version, the Exploratory Factor Analysis (EFA) was conducted on an independent sample (participants from Study 1). Based on the EFA results, the measure was further refined to 14 items organized into two subscales. Subsequently, Confirmatory Factor Analysis (CFA) was performed to evaluate the adequacy of the proposed factor structure (participants from Study 2). Both a two-factor model and a bifactor model were tested and demonstrated acceptable fit, indicating their suitability for different scoring and interpretive purposes. Finally, relationships between a tendency toward depersonalization and depressive symptoms were examined using correlation and regression analyses.

### Measures

Depersonalization Mechanism Scale (short version) includes initial 18 items designed to assess a tendency to depersonalization (3). It consists of two subscales: *Detachment* and *Emotional Numbness*. Participants rate items using a 5-point Likert scale, where 0 is “never” and 4 is “always”. Total score is created by summing up the responses (ranging from 0 to 72), with higher score indicating higher level of depersonalization tendency. The final version of the scale consists of 14 items organized into two subscales: *Detachment* and *Emotional Numbness.* The scoring remains unchanged, with the total score ranging from 0 to 56, where higher score indicated a greater tendency towards depersonalization. The instrument has high internal consistency, with Cronbach’s α above.90.
Patient Health Questionnaire (PHQ-9) (22) is a 9-item questionnaire designed for depression screening. Participants evaluate the severity of depressive symptoms from the previous two weeks on a 4-point scale (0, “not at all”; 3, “every day”). Scores range from 0 to 27, signifying the severity of depressive symptoms (form minimal to severe).In this study, Cronbach’s *α* of the Polish version is.89.
Occupational Depression Inventory (23) is a 9-item questionnaire designed for assessment of work-related depressive symptoms. Questions correspond to DSM-5 diagnostic criteria for depression and focus of the anhedonia, depressed mood, sleep alterations, fatigue/loss of energy, appetite alterations, feelings of worthlessness, cognitive impairment, psychomotor alterations, and suicidal ideation. It was inspired by PHQ-9 (22) and enables a provisional diagnosis of job-ascribed depression. Participants rate their experiences of work-related depressive symptoms from the previous two weeks on a 4-point scale (0, "never"; 3, "nearly every day"). The Polish version of ODI presents high internal consistency, with Cronbach’s α coefficients ranging from.90 to.94 (24).

## Results

All analyses were conducted using R (version 4.5.2; R Core Team, 2025) and JASP (0.95.4; 2025). Exploratory factor analysis (EFA) was performed using the `psych` package (25). Confirmatory factor analysis (CFA) was estimated using the `lavaan` package (26). Reliability indices and additional psychometric indicators were computed using the `semTools` package (27) and `psych`. Polychoric correlation matrices were obtained using `psych::polychoric()`.

Descriptive statistics were calculated for all variables (**Table 1**). Although Kolmogorov-Smirnov tests indicated significant deviations from normality, a parametric correlation (*r*) was used. This approach is robust to non-normality given the sample sizes and the absence of outliers, as supported by the Central Limit Theorem. Significance level was set at standard *α*, 0.05.

**Table 1:** Descriptive statistics.

Method	Subscale	*M*	*Me*	*SD*	*Sk.*	*Kurt.*	*Min.*	*Max*	*W*	*p*
DMS	Depersonalization	27.17	27	17.67	.29	-0.50	0	80	0.06	<.001
	Emotional numbness	15.55	16	9.20	.12	-0.52	0	40	.074	<.001
	Detachment	11.62	11	9.57	.50	-0.59	0	40	.112	<.001
PHQ	-––-	9.29	9	6.94	.47	-0.57	0	27	.090	<.001
ODI	-––-	7.23	4	7.65	.75	-0.59	0	27	.205	<.001

M, mean; Me, median; SD, standard deviation; Sk., skewness.; Kurt., kurtosis; Min and Max., minimum and maximum value; W, Kołmogorov-Smirnov test; p, significance level; DMS, Depersonalization Mechanism Scale; ODI, Occupational Depression Inventory; PHQ, Patient Health Questionnaire.

### Scale reduction process

Prior to factor extraction, the suitability of the data for factor analysis was confirmed. The overall KMO measure of sampling adequacy was MSA, .956, well above the recommended threshold ≥.80 (28), with item-level MSA values ranging from.919 to.981. Bartlett’s test of sphericity was statistically significant, χ² (820), 10,513.07, p <.001 (29), further supporting the appropriateness of the analysis. Parallel analysis, corroborated by the eigenvalue > 1 criterion and visual inspection of the scree plot, indicated a two-factor solution. Following Promax rotation, the sum of squared loadings were 12.787 (37.6%) and 7.003 (20.6%), respectively, with total explained variance remaining at 58.2%.

Through an iterative item elimination procedure, seven items were removed. Items were excluded on the basis of two criteria applied sequentially (1): low primary factor loadings, with elimination proceeding iteratively from the weakest-loading items, and (2) cross-loadings, whereby items exhibiting substantial loadings on more than one factor were removed to ensure factorial clarity and discriminant validity of the resulting subscales. Factor loadings of the retained items ranged from.543 to.971 on Factor 1 and from.574 to.885 on Factor 2. In addition to factor loadings, the item reduction procedure involved removing redundant items, retaining those with clearer and more natural wording, and excluding items specifically related to sensory experiences. A detailed description of the decision-making process is provided in the Supplementary Materials (**Supplementary Table 1**).

### Exploratory factor analysis

Given the ordinal, Likert-type nature of item responses, EFA was conducted on a polychoric correlation matrix, which provides more accurate estimates of latent structure for ordinal indicators than Pearson correlations (30, 31). Factor extraction was performed using the minimum residuals (minres) method. Promax (oblique) rotation was applied throughout, consistent with the theoretical expectation that the two factors of depersonalization would correlate. The same rotation method was used at all stages of item reduction to ensure analytic consistency.

Prior to extraction, the suitability of the data was evaluated using the Kaiser–Meyer–Olkin (KMO) measure of sampling adequacy, with a criterion of ≥.80 (28) and Bartlett’s test of sphericity (29). The number of factors to retain was determined using three convergent criteria (1): the eigenvalue > 1 (32) (2); visual inspection of the scree plot (33); and (3) parallel analysis (34). Parallel analysis was given primary weight in the retention decision, as it is considered one of the most accurate factor retention methods currently available (35, 36).

Prior to factor extraction, multivariate normality was assessed using Mardia’s test, which indicated significant multivariate skewness and kurtosis (Skewness, 54.05, χ² (560), 1837.68, *p* <.001; Kurtosis statistic, 316.07, *z*, 31.07, *p* <.001), confirming that a normality-robust estimation method was appropriate.

The item reduction procedure for the 14-item solution followed an iterative approach applied to the 18-item pool retained after the initial reduction from the original 41-item DMS. Items were evaluated sequentially based on two criteria: 1) the magnitude of the primary factor loading, and 2) the difference between the primary and cross-loadings, with preference for removing items showing the lowest primary loadings combined with the smallest loading differences (i.e., lowest discriminability between factors). Following this procedure, Items 1, 3, 4, and 12 were excluded. Item 1 and Item 4 showed high inter-item redundancy with the remaining Detachment items and added minimal unique variance. Item 3 demonstrated a relatively low primary loading with insufficient separation from the alternative factor. Item 12 was excluded at this stage due to marginal discriminant contribution relative to the remaining Emotional Numbness items.

**Figure 1:**
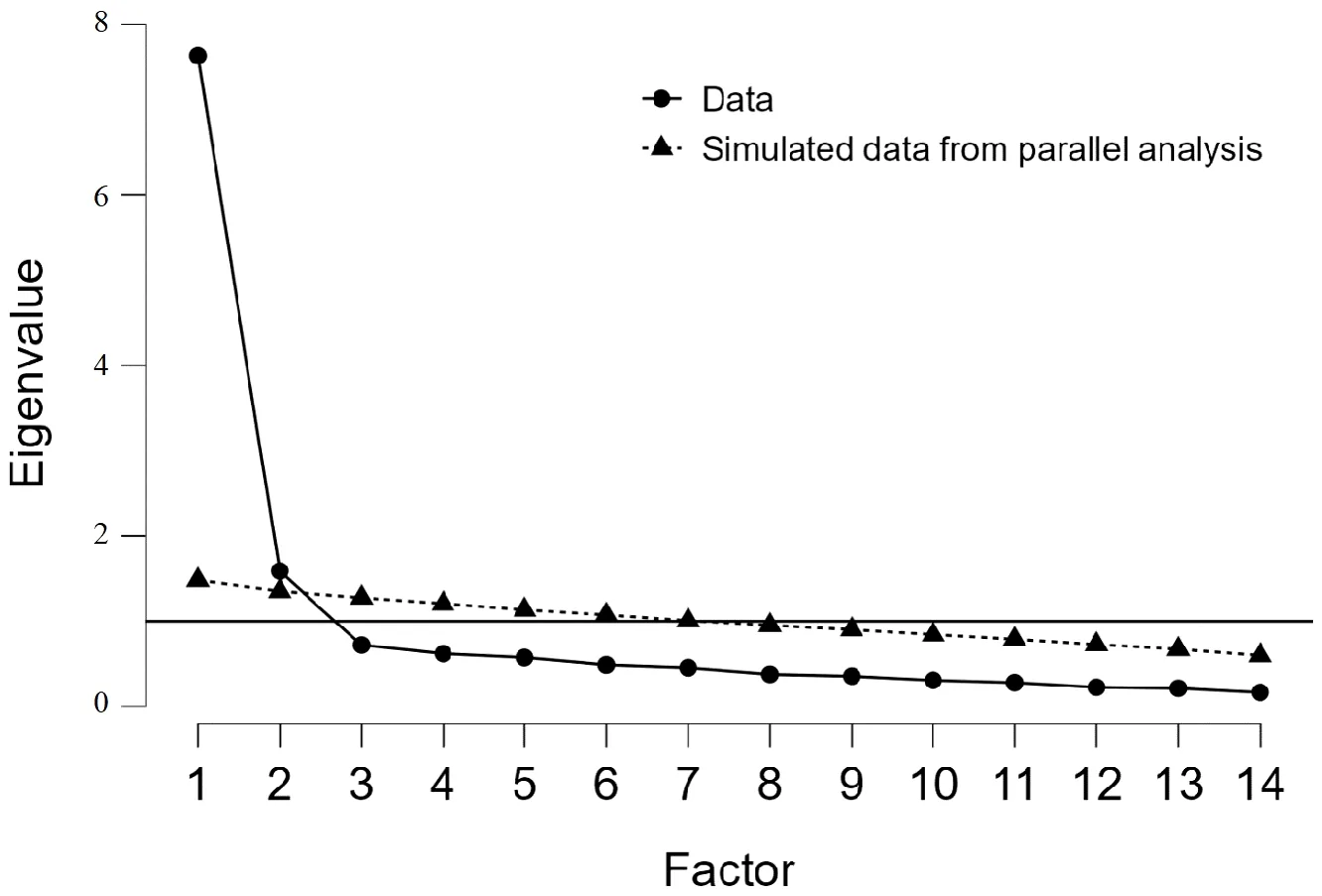
Scree plot of eigenvalues for the identification of two factors.

The adequacy of the 14-item data for factor analysis was confirmed by KMO, .931 and Bartlett’s test of sphericity, χ² (91), 1900.75, *p* <.001. EFA with minres extraction and Promax rotation was conducted on the polychoric correlation matrix. Parallel analysis and scree plot (**Figure 1**) inspection both supported the retention of two factors. The eigenvalue-greater-than-one criterion also yielded two factors in the final 14-item solution (Factor 1: eigenvalue, 7.241, 51.7% of variance; Factor 2: eigenvalue, 1.197, 8.5% of variance; total variance explained, 60.3%). The two-factor solution was therefore adopted, consistent across all three retention criteria and with the established theoretical structure of the DMS.

The model chi-square was significant, χ² (64), 167.49, *p* <.001, as expected given the sample size. The first factor, labelled *Detachment*, comprised 8 items (Items 2, 5, 6, 7, 8, 16, 17, 18) with primary loadings ranging from.552 to.938. The second factor, labelled *Emotional Numbness*, comprised 6 items (Items 9, 10, 13, 14, 15, 20) with loadings ranging from.684 to.846. All primary loadings exceeded.50, and no cross-loadings exceeded.30 (**Table 2**), satisfying the criterion that the difference between primary and cross-loadings be at least.20 (37). The two factors were positively correlated (*r*, .61), supporting the use of oblique rotation and motivating bifactor modelling in the subsequent CFA stage.

**Table 2:** EFA factor loadings — 14-item solution (promax rotation).

Item	Item content	Factor 1 (detachment)	Factor 2 (emotional numbness)	Uniqueness
16	I have the experience of not being fully connected to my body.	.938	—	.254
5	I have trouble fully feeling my own body.	.896	—	.349
6	I have an impression as if my emotions and thoughts were not coherent with myself.	.867	—	.282
7	My surroundings seem far away and unclear, as if I were looking at it through a fog.	.733	—	.380
2	My thoughts seem to float uncontrollably.	.706	—	.413
17	When I experience something difficult, everything seems so “flat” and “faded”.	.691	—	.367
18	I have the experience of watching my life from the distance.	.690	—	.394
8	I catch myself being so invested in daydreaming that it interferes with my day-to-day life.	.552	—	.546
15	I feel emotionally distanced from others.	—	.846	.363
14	I have so little mental energy that I do the bare minimum when I interact with others.	—	.784	.362
20	I avoid contact with other people.	—	.735	.387
10	When things get hard, I become more and more cynical, relationships with others become less meaningful.	—	.699	.473
9	I do my business and I don’t want to be bothered.	—	.693	.557
13	It seems that people around me are particularly difficult.	—	.684	.436

Minimum residuals (minres) extraction with Promax rotation; polychoric correlation matrix. Loadings <.30 are suppressed. Factor 1 eigenvalue, 7.241 (51.7% variance); Factor 2 eigenvalue, 1.197 (8.5% variance); total variance explained, 60.3%.

### Confirmatory factor analysis

CFA was performed on the second, independent sample (*N*, 367) to test the factor structure identified in the EFA. Two models were specified and compared: a correlated two-factor model (CFA_v1) and a bifactor model (CFA_BIF), both using all 14 items.

All items were treated as ordered categorical variables prior to CFA estimation. The Weighted Least Squares Mean and Variance Adjusted (WLSMV) estimator was applied throughout, as it is appropriate for ordinal indicators and robust to violations of multivariate normality (38, 39). Estimation was based on polychoric correlations, consistent with the EFA phase.

Model fit was evaluated using multiple indices (40, 41): the Comparative Fit Index (CFI) and Tucker–Lewis Index (TLI), with values ≥.95 indicating acceptable fit; the Root Mean Square Error of Approximation (RMSEA) with its 90% confidence interval, with values <.08 indicating acceptable and <.06 indicating close fit; and the Standardized Root Mean Square Residual (SRMR), with values <.08 acceptable. Because the chi-square statistic is sensitive to sample size (42, 43), primary interpretive weight was given to the incremental and absolute fit indices rather than χ² alone.

Convergent validity was assessed using Average Variance Extracted (AVE), with a criterion of ≥.50 (44) and Composite Reliability (CR), with a criterion of ≥.70 (45). Discriminant validity was assessed using the Heterotrait–Monotrait (HTMT) ratio of correlations (46), with thresholds of ≤.85 (stringent) and ≤.90 (permissible).

To further examine the structure of the scale and address the high interfactor correlation, a bifactor model was specified and compared to the correlated two-factor solution. McDonald’s omega (ω) based on the factor model was reported as the primary reliability index, alongside Cronbach’s α for comparability.

### Model 1: two-factor model (CFA_v1)

The first model specified two correlated latent factors: *Detachment* (C1; 8 items: 2, 5, 6, 7, 8, 16, 17, 18) and *Emotional Numbness* (C2; 6 items: 9, 10, 13, 14, 15, 20). The model yielded: χ² (76), 330.69, *p* <.001; CFI, .981; TLI, .978; RMSEA, .096 (90% CI [.085,.106]), *p*(RMSEA ≤.05) <.001; SRMR, .038. The CFI and TLI values exceeded the.95 acceptable threshold, indicating good fit on incremental indices (**Table 3**). The SRMR of.038 indicated low residuals. The RMSEA of.096, however, exceeded both the conservative (.06) and acceptable (.08) cutoffs, suggesting some degree of model misfit, potentially related to the complexity of item-level covariance structure in this scale with the WLSMV estimator. The model fit is therefore characterized as mixed: good on incremental indices, but insufficient on RMSEA.

**Table 3:** CFA model fit indices.

Index	CFA_v1 (2-factor, 14 items)	CFA_BIF (bifactor, 14 items)
χ²(df)	330.69 (76)	183.51 (63)
p	<.001	<.001
CFI	.981	.991
TLI	.978	.987
RMSEA	.096	.072
RMSEA 90% CI	[.085,.106]	[.060,.085]
p(RMSEA ≤.05)	<.001	.002
SRMR	.038	.025

WLSMV estimator. Formal chi-square difference testing is not available for WLSMV; model comparison is based on descriptive differences in fit indices. AIC and BIC are not provided as WLSMV does not yield likelihood-based information criteria.

Both factors demonstrated strong convergent validity: *Detachment* (C1): AVE, .763, CR, .963, α, .948, ω, .963; *Emotional Numbness* (C2): AVE, .664, CR, .922, α, .903, ω, .922 (**Table 4**). AVE values substantially exceeded the.50 threshold and CR values exceeded.90 for both factors. Discriminant validity was supported by HTMT, .812 (<.85 stringent threshold), indicating that the two factors are empirically distinct despite their substantial correlation (*r*, .825, SE, .018, *z*, 45.77, *p* <.001).

**Table 4:** Reliability and convergent validity indices.

Model	Factor	n items	AVE	CR	α	ω
CFA_v1	C1, Detachment	8	.763	.963	.948	.963
CFA_v1	C2, Emotional Numbness	6	.664	.922	.903	.922
CFA_BIF	G, General Depersonalization	14	.627	.958	.952	.958
CFA_BIF	C1, Detachment (specific)	8	.029	.016	.948	.016
CFA_BIF	C2, Emotional Numbness (specific)	6	.231	.636	.903	.636

WLSMV estimator; polychoric correlation matrix. AVE, Average Variance Extracted; CR, Composite Reliability; α, Cronbach’s alpha; ω, McDonald’s omega. HTMT (CFA_v1): C1–C2, .812 (<.85 stringent threshold). HTMT not applicable for CFA_BIF (orthogonal factors by design).

Standardized factor loadings, standard errors, *z*-values, and item-level R² are reported in Supplementary Materials (**Supplementary Table 2**).

### Model 2: bifactor model (CFA_BIF)

To assess the extent to which DMS subscales capture variance beyond a general depersonalization factor, a bifactor model was specified with a general factor (G) loading on all 14 items and two orthogonal specific factors — Detachment (C1; 8 items) and Emotional Numbness (C2; 6 items) — with all interfactor covariances constrained to zero, consistent with standard bifactor parameterization (47). Estimation followed the same procedure as CFA (WLSMV; polychoric correlations; ordered categorical items). Beyond standard fit and reliability indices, the bifactor solution was evaluated using omega hierarchical (ωh; criterion: ≥.50), omega subscale (ωs; criterion: ≥.50), Explained Common Variance (ECV_G; criterion: ≥.70), and Percent of Uncontaminated Correlations (PUC; criterion: >.80), computed from the standardized loading matrix following Rodriguez et al. (48).

The bifactor model specified a general factor (G) loading on all 14 items alongside two specific factors: *Detachment* (C1; 8 items) and *Emotional Numbness* (C2; 6 items). All three factors were constrained to be mutually orthogonal (G ~~ 0C1; G ~~ 0C2; C1 ~~ 0C2), consistent with standard bifactor parameterization (46). The model yielded: *χ² (*63), 183.51, *p* <.001; CFI, .991; TLI, .987; RMSEA, .072 (90% CI [.060,.085]), *p*(RMSEA ≤.05), .002; SRMR, .025 (**Table 3**).

The bifactor model showed improved fit over CFA_v1 across all indices: ΔCFI, +.010, ΔTLI, +.009, ΔRMSEA, −.024, ΔSRMR, −.013. The RMSEA of.072 remained above the conservative.06 cutoff but fell within the commonly accepted <.08 range, and the lower bound of its 90% CI reached.060. The SRMR of.025 indicated very low residuals.

The general factor demonstrated strong reliability and convergent validity: AVE, .627, CR, .958, α, .952, ω, .958. The specific factors, however, showed markedly reduced indices after partialling out the general factor variance: C1 (AVE, .029, CR, .016, ω, .016) and C2 (AVE, .231, CR, .636, ω, .636) (**Table 4**). This pattern indicates that the general depersonalization factor captures the vast majority of common variance across items, with the specific factors accounting for minimal unique reliable variance beyond G. The high Cronbach’s α values observed for the specific factors (α_C1_, .948; α_C2_, .903) reflect item-level covariance attributable to the general factor rather than specific-factor variance and should not be interpreted as indicators of specific-factor reliability in the bifactor context (49). HTMT is not applicable to bifactor models due to the imposed orthogonality constraint between all factors. Standardized factor loadings for G, C1, and C2, including standard errors, *z*-values, and item-level R², are reported in Supplementary Materials (**Supplementary Table 3**).

### Bifactor-specific psychometric indices

To further examine the internal structure of the scale and address the concern regarding the interfactor correlation observed in CFA_v1 (*r*, .825), bifactor-specific indices recommended by Rodriguez et al. (47) and Reise (46) were computed for CFA_BIF: omega hierarchical (ωh), omega subscale (ωs), Explained Common Variance (ECV), and Percent of Uncontaminated Correlations (PUC). These indices are summarized in **Table 5**.

Omega hierarchical was ωh, .911, substantially exceeding the ≥.50 criterion (47), indicating that 91.1% of the variance in DMS total scores is attributable to the general depersonalization factor G. This finding provides strong psychometric support for the use of a total DMS score as a reliable and valid composite.

The general factor accounted for ECV_G_, .844 of total common variance, well above the ≥.70 criterion (48), confirming that G dominates the common variance among items. The specific factors contributed only negligible proportions of common variance: ECV_C1_ (Detachment), .022 and ECV_C2_ (Emotional Numbness), .133.

Omega subscale values were markedly low after partialling out general factor variance: ωs_C1_, .002 (Detachment) and ωs_C2_, .311 (Emotional Numbness), both falling below the ≥.50 criterion, indicating that the specific factors carry insufficient unique reliable variance to justify independent subscale scoring within a bifactor framework. PUC, .527 fell below the >.80 criterion, indicating that only 52.7% of item pairs belong to different subscales and are thus correlated solely through G. It should be noted that this low PUC value is a structural feature of the scale - resulting from two subscales of unequal size (8 and 6 items) rather than an analytic artefact - and does not invalidate the other indices. Nonetheless, as noted by Rodriguez et al. (48), ωh and ECV_G_ should be regarded as conservative estimates under these conditions and interpreted accordingly.

### Model comparison

The bifactor model demonstrated superior global fit relative to the correlated two-factor model (**Tables 3**, **5**). However, the near-zero specific-factor AVE and ω in CFA_BIF indicate that the subscale scores (Detachment and Emotional Numbness) lack sufficient reliable specific-factor variance to be meaningfully interpreted or used independently when bifactor scoring is applied. In contrast, the two-factor oblique model (CFA_v1) provides strong and theoretically coherent subscale scores, with excellent AVE, CR, ω, and discriminant validity (HTMT, .812). Taken together, the two-factor correlated model is recommended as the primary scoring model for the 14-item DMS. The bifactor results additionally confirm the psychometric integrity of the total DMS score (ωh, .911), which is thus recommended for research purposes requiring a single composite measure. Both scoring approaches — total score and two subscales — are psychometrically justified for different research purposes (50). A comparison of fit indices across both models is presented in **Table 3** and reliability and convergent validity indices for both models are presented in **Table 5**.

**Table 5:** Bifactor indices for CFA_BIF (14-item bifactor model).

Index	Value	Criterion
ωh — omega hierarchical	.911	≥.50
ωs C1 — Detachment	.002	≥.50
ωs C2 — Emotional Numbness	.311	≥.50
ECV — G (general factor)	.844	≥.70
ECV — C1 (Detachment)	.022	—
ECV — C2 (Emotional Numbness)	.133	—
PUC	.527	>.80

ωh, omega hierarchical; ωs, omega subscale; ECV, Explained Common Variance; PUC, Percent of Uncontaminated Correlations. Indices computed following Rodriguez et al. (2016) and Reise (2012). Low PUC (.527) indicates that ωh and ECV_G should be interpreted with caution. WLSMV estimator; polychoric correlation matrix.

### Associations with depressive symptoms

Correlations between depersonalization and depressive symptoms are presented in **Table 6**. Depersonalization and its subscales were strongly and positively correlated with depressive symptoms in both samples (*p* < 0.001).

**Table 6:** Pearson correlations between depersonalization and depressive symptoms.

Measure	Detachment	Emotional numbness	Depersonalization
PHQ	.68***	.62***	.71***
ODI	.60***	.65***	.66***

*Note.* *** p <.001. PHQ-9, Patient Health Questionnaire-9 (general depressive symptoms); ODI, Occupational Depression Inventory (work-related depressive symptoms).

Two separate multiple regression models were estimated, with Detachment and Emotional Numbness as predictors and depressive symptoms as the outcome variable - once for PHQ-9 and once for ODI, given the distinct conceptual focus of each instrument. Full regression coefficients are presented in **Table 7**.

**Table 7:** Regression coefficients: DMS subscales predicting depressive symptoms.

Measure	Variables	*b*	*SE*	*β*	*t*	*p*	95% CI	*F*	adj. R^2^
PHQ	Constant	2.03	.44		4.57	<.001	[1.15, 2.90]	236.30***	.500
	Detachment	.43	.04	.47	10.43	<.001	[.35,.51]		
	Emotional numbness	.35	.05	.29	6.48	<.001	[.24,.46]		
ODI	Constant	1.53	.95		1.61	.112	[-.36, 3.42]	38.95***	.436
	Detachment	.23	.10	.26	2.25	.027	[.03,.42]		
	Emotional numbness	.52	.13	.45	3.91	<.001	[.26,.78]		

***p <.001. PHQ-9, Patient Health Questionnaire-9; ODI, Occupational Depression Inventory. b, unstandardized coefficient; SE, standard error; β, standardized coefficient; 95% CI for B.

For PHQ-9 (general depressive symptoms), the model was statistically significant, *F (2*, 472), 236.30, *p* <.001, adj. R², .500, indicating that the two DMS subscales jointly account for 50.0% of the variance in general depressive symptoms. Both predictors were significant: Detachment (*b*, .43, *SE*, .04, *β*, .47, *t*, 10.43, *p* <.001, 95% CI [.35,.51]) and Emotional Numbness (*b*, .35, *SE*, .05, *β*, .29, *t*, 6.48, *p* <.001, 95% CI [.24,.46]).

For ODI, the model was also significant, *F (2*, 201), 38.95, *p* <.001, adj. R², .436. Both predictors were significant: Detachment (*b*, .23, SE, .10, *β*, .26, *t*, 2.25, *p*, .027, 95% CI [.03,.42]) and Emotional Numbness (*b*, .52, SE, .13, *β*, .45, *t*, 3.91, *p* <.001, 95% CI [.26,.78]).

Notably, the pattern of relative predictor weights differed across outcomes. For PHQ-9, Detachment was the stronger predictor (*β*, .47 vs. *β*, .29), whereas for ODI, Emotional Numbness contributed more uniquely (*β*, .45 vs. *β*, .26). This dissociation suggests that the experience of emotional blunting and interpersonal withdrawal may be particularly characteristic of work-related depression, while perceptual and cognitive detachment shows a stronger association with general depressive symptomatology.

## Discussion

The present study aimed to develop and validate a shortened version of the Depersonalization Mechanism Scale (DMS) and to examine the association between depersonalization and depressive symptoms in a non-clinical sample. The findings indicate that the 14-item version of the DMS preserves the two-factor structure identified in the original scale, comprising the dimensions of Detachment and Emotional Numbness. Both subscales and the total score, demonstrated excellent internal consistency. The bifactor analysis suggests the presence of a strong general depersonalization factor, indicating that both specific dimensions may reflect a broader underlying construct. Furthermore, depersonalization was strongly associated with depressive symptoms, replicating findings obtained with the full version of the instrument (3). Together, these results provide support for the psychometric validity of the shortened scale and its potential utility in future research.

An important issue concerns the strong association between the Detachment and Emotional Numbness subscales. Although the results supported the proposed two-factor structure, the magnitude of the correlation suggests that the two dimensions share considerable common variance. The high correlation raises questions regarding discriminant validity and suggests that the distinction between these dimensions should be interpreted cautiously. The results of the bifactor analysis indicate that the overlap between the two subscales is largely driven by a general depersonalization factor. Future studies should further examine whether the distinction between Detachment and Emotional Numbness provides incremental explanatory value beyond the general depersonalization construct.

The observed associations between depersonalization and depressive symptoms is consistent with previous findings demonstrating a close relationship between these phenomena in both clinical (18) and non-clinical (3) populations. In the present study, higher levels of depersonalization were associated with greater severity of depressive symptoms measured by two independent scales (PHQ and ODI). The results do not permit conclusions regarding the direction or causal nature of this relationship. Depersonalization may contribute to depressive experiences, depressive symptoms may increase the likelihood of depersonalization, or both constructs may arise from shared psychological or environmental factors. Michal et al. (18) provide evidence consistent with a causal relationship between depersonalization and depression severity in a clinical sample. However, longitudinal research is needed to investigate whether depersonalization represents a correlate, consequence, or risk factor for depressive symptomatology in non-clinical populations.

Several limitations should be considered when interpreting the present findings. First, the cross-sectional design precludes conclusions regarding cause-and-effect relationships between depersonalization and depressive symptoms. Second, the exclusive reliance on self-report measures may have increased susceptibility to response biases and common-method variance. Third, the absence of external validation measures and clinical diagnostic assessments restricts conclusions regarding the clinical significance of the observed associations. Fourth, test–retest reliability was not assessed, and therefore the temporal stability of the shortened scale remains to be established. Fifth, the relatively small, predominantly young samples, recruited online and consisting exclusively of Polish participants, may limit the generalizability of the findings to other populations and cultural contexts. Finally, because both depersonalization and depressive symptoms involve aspects of emotional disengagement, some degree of conceptual overlap between the measured constructs cannot be excluded.

The limitations of the present study suggest several directions for future research. In particular, longitudinal research is needed to assess the temporal stability of depersonalization and clarify its associations with subsequent mental health outcomes. Multimethod approaches integrating self-report measures with clinical assessments and behavioral indicators of emotion regulation may provide a more comprehensive understanding of depersonalization and its associations with other psychiatric and psychological constructs. Qualitative studies, such as semi-structured interviews, may further complement these efforts by offering insights into the subjective experience and temporal dynamics of depersonalization. Further studies should also evaluate measurement invariance across diverse sociodemographic and cultural groups. Another important research direction is to investigate depersonalization independently of depressive symptoms and to identify determinants of depersonalization in non-clinical populations. In this context, it would be valuable to compare non-clinical individuals without depressive symptoms who differ in the severity of depersonalization and examine these groups with respect to various individual and contextual factors.

In conclusion, the shortened DMS demonstrated satisfactory psychometric properties and retained the theoretical structure of the original instrument. Its brief format makes it suitable for large-scale studies investigating depersonalization alongside multiple psychological constructs. Depersonalization was strongly associated with depressive symptoms, although the causal nature of this relationship remains to be established. The present findings support the use of the shortened DMS in future research and contribute to a better understanding of depersonalization as a phenomenon relevant to emotional functioning.

## Data Availability

The raw data supporting the conclusions of this article will be made available by the authors, without undue reservation.
