# Supplementary material for: Depersonalization and depressive symptoms in two online samples: development and initial validation of a short Depersonalization Mechanism Scale
Source: Front Psychiatry. 2026 Aug 5;17:1892456. doi: 10.3389/fpsyt.2026.1892456 (PMC13488167; doi:10.3389/fpsyt.2026.1892456)
Supplement: Supplementary file 1 [file Table1.pdf]

Table S1

DMS item reduction analysis decisions and justifications (from 41 to 18 items).

		Factor Loading		
**Subscale**	**Item**	**Detachment**	**Emotional Numbness**	**Justification for Item Removal**
DE	1. I have trouble recalling happy memories, even though I know I have them	<0.5 or cross loading		Initial reduction after rotation.
DE	2. I have the experience of being outside of my body/ watching myself from the distance	0.93	—	Substantial content overlap with Item 8, which was retained because it demonstrates a higher factor loading and more effectively captures the experience of detachment from one's body.
DE	3. Smells do not evoke neither pleasant nor unpleasant feelings.	0.58	—	The item refers to sensory experiences that are not considered a core feature of depersonalization; reliability increase if deleted.
DE	4. It seems as if my thoughts are outside of my control, as if they are not consistent with the rest of my experiences.	0.69	—	Substantial content overlap with Item 10 which was retained because it demonstrates a similar factor loading and better captures the lack of control over depersonalization experiences.
DE	5. I go through my day as if I am on autopilot and at times, I catch myself that I don't remember what was happening	<0.5 or cross loading		Initial reduction after rotation.
DE	6. My body feels strange	0.88	—	Substantial content overlap with Items 8, 13, and 30, which demonstrates comparable factor loadings and greater specificity.
EN	7. Other people seem unhelpful and ungrateful	—	0.68	Substantial content overlap with Item 24, which was retained because it captures a broader range of relational difficulties despite a lower factor loading.

DE	8. I have the experience of being out of touch with my body	0.94	—	RETAINED
DE	9. In difficult times, I have the experience of being detached from my emotions	0.67	—	Substantial content overlap with Item 23, which was retained because it more directly captures the respondent's subjective experience despite a lower factor loading.
DE	10. My thoughts seem to float uncontrollably	0.67	—	RETAINED
EN	11. I try my best even though I am exhausted and yet nobody appreciates it	—	0.72	RETAINED
DE	12. I have the experience of being “spaced out”	0.63	—	RETAINED
DE	13. I have trouble fully feeling my own body	0.87	—	RETAINED
DE	14. I have an impression as if my emotions and thoughts were not coherent with myself	0.73	—	RETAINED
EN	15. I wish I had more control over my emotions	—	0.65	Conceptually distinct from the remaining Emotional Numbness items, as it primarily reflects beliefs about emotions rather than subjective experiences of emotional detachment or numbness during stressful situations.
DE	16. My surroundings seem far away and unclear, as if I were looking at it through a fog	0.84	—	RETAINED
DE	17. I catch myself being so invested in daydreaming that it interferes with my day-to-day life	0.67	—	RETAINED
EN	18. I do my business and I don’t want to be bothered	—	0.89	RETAINED

EN	19. When things get hard, I become more and more cynical, relationships with others become less meaningful	—	0.58	RETAINED
EN	20. I only engage in what is necessary	—	0.63	Substantial content overlap with Item 27, which was retained because it more directly captures reduced emotional capacity despite a lower factor loading.
DE	21. I find myself treating others in an impersonal and almost automatic manner	<0.5 or cross loading		Initial reduction after rotation.
DE	22. I start distancing myself from everyone	—	0.80	Substantial content overlap with Item 28, which was retained because it places greater emphasis on emotional distance from others, a more central feature of depersonalization, despite a lower factor loading.
EN	23. I feel emotionally detached from my surroundings	—	0.61	RETAINED
EN	24. It seems that people around me are particularly difficult	—	0.62	RETAINED
DE	25. I am so invested in fantasies that I feel like I am experiencing them for real	0.72	—	Substantial content overlap with Item 17, which was retained because it better reflects the functional disruptions associated with extensive daydreaming, despite a lower factor loading. The present item is more strongly related to imagination and is therefore less central to the construct.
DE	26. I realize how disconnected I am	0.54	—	Substantial content overlap with Item 12, which was retained because it captures the subjective unease associated with depersonalization more fully; additionally, the present item shows a comparatively lower factor loading than other items within the factor.
EN	27. I have so little mental energy that I do the bare minimum when I interact with others	—	0.57	RETAINED
EN	28. I feel emotionally distanced from others	—	0.75	RETAINED

DE	29. When I look in the mirror it is like I am looking at a stranger – I know it is me, but I don't feel emotionally connected to my reflection	0.89	—	Substantial content overlap with Item 30, which was retained because it demonstrated a higher factor loading and more broadly captures experiences of bodily disconnection.
DE	30. I have the experience of not being fully connected to my body	0.95	—	RETAINED
DE	31. When I experience something difficult, everything seems so "flat" and "faded"	0.63	—	RETAINED
DE	32. I realize that I have no feelings in situations when I would normally feel something	0.54	—	This item shows a comparatively lower factor loading than other items within the factor.
DE	33. I have the experience of watching my life from the distance	0.68	—	RETAINED
DE	34. The food has less distinct flavour	0.69	—	The item refers to sensory experiences that are not considered a core feature of depersonalization. Its content may be more closely related to sensory sensitivity than to a tendency toward depersonalization.
DE	35. I have the experience of having a blank space in my head when I talk	<0.5 or cross loading		Initial reduction after rotation.
DE	36. When someone asks me a question, I feel as if I am answering automatically	<0.5 or cross loading		Initial reduction after rotation.
DE	37. Familiar voices appear strange and peculiar.	0.97	—	The item refers to sensory experiences that are not considered a core feature of depersonalization. Additionally, it differs from the remaining items in its level of specificity, focusing on a narrow set of phenomena related to familiar sensory stimuli rather than broader experiences of depersonalization.

EN	38. I start to distance myself and care very little about everything	<0.5 or cross loading		Initial reduction after rotation.
DE	39. The fantasy world is a kind of escape from hard reality	<0.5 or cross loading		Initial reduction after rotation.
EN	40. When something particularly hard happens to me, I need a lot of time to gain balance again	—	0.72	The item extends beyond the immediate experiences assessed by the remaining items by focusing on the duration of recovery. As such, it may be more closely related to reduced capacity than to the core construct represented by this factor.
EN	41. I avoid contact with other people	—	0.62	RETAINED

Note. DE – Detachment; EN – Emotional Numbness.
