# Supplementary material for: Depersonalization and depressive symptoms in two online samples: development and initial validation of a short Depersonalization Mechanism Scale
Source: Front Psychiatry. 2026 Aug 5;17:1892456. doi: 10.3389/fpsyt.2026.1892456 (PMC13488167; doi:10.3389/fpsyt.2026.1892456)
Supplement: Supplementary file 2 [file Table2.pdf]

Table S2
Standardized Factor Loadings for the Two-Factor Model (CFA_v1), N = 367

Item	Item content (abbreviated)	Factor	λ	SE	z	R^2
16	No sense of connection with own body	*C1*	.885	.013	69.33	.783
5	Not fully feeling own body	*C1*	.899	.012	77.46	.808
6	Emotions and thoughts feel incoherent	*C1*	.877	.013	68.63	.770
7	Looking at the world through fog	*C1*	.879	.014	64.06	.773
17	Everything looks faded and flat	*C1*	.894	.013	71.30	.799
18	Observing one's life from a distance	*C1*	.888	.012	72.80	.789
2	Thoughts drifting beyond control	*C1*	.846	.016	52.61	.715
8	Becoming lost in fantasies	*C1*	.818	.018	45.48	.670
15	Emotional distance from others	*C2*	.824	.019	43.20	.679
14	Limiting interactions to minimum	*C2*	.876	.016	53.34	.767
20	Avoiding contact with others	*C2*	.790	.022	35.55	.624
10	Becoming cynical; relationships lose meaning	*C2*	.855	.018	48.68	.731
13	Others seem particularly difficult	*C2*	.806	.020	39.47	.649
9	Preoccupied with own affairs; wanting to be left alone	*C2*	.733	.026	28.08	.537
r	C1–C2 interfactor correlation	—	.825	.018	45.77	—

Note. C1 = Detachment; C2 = Emotional Numbness. All items treated as ordered categorical; WLSMV estimator; polychoric correlation matrix. λ = standardized factor loading; SE = standard error; R^2 = proportion of item variance explained by the factor model. Interfactor correlation ($r = .825$, SE = .018, $z = 45.77$, $p < .001$) estimated under oblique rotation (Promax-consistent parameterization). All factor loadings significant at $p < .001$.
