# Supplementary material for: Depersonalization and depressive symptoms in two online samples: development and initial validation of a short Depersonalization Mechanism Scale
Source: Front Psychiatry. 2026 Aug 5;17:1892456. doi: 10.3389/fpsyt.2026.1892456 (PMC13488167; doi:10.3389/fpsyt.2026.1892456)
Supplement: Supplementary file 3 [file Table3.pdf]

Table S3

Standardized Factor Loadings for the Bifactor Model (CFA_BIF), N = 367

Item	Item content (abbreviated)	Sp.	General factor (G)			Specific factor			R^2
			λ	SE	z	λ	SE	Z	
16	No sense of connection with own body	*C1*	.881	.013	65.31	.119	.044	2.68*	.789
5	Not fully feeling own body	*C1*	.898	.012	75.24	.029	.056	0.51	.808
6	Emotions and thoughts feel incoherent	*C1*	.870	.017	50.85	.288	.049	5.89***	.839
7	Looking at the world through fog	*C1*	.881	.014	63.53	−.033	.049	−0.67	.776
17	Everything looks faded and flat	*C1*	.900	.014	66.15	−.157	.049	−3.17**	.834
18	Observing one's life from a distance	*C1*	.896	.014	63.17	−.196	.053	−3.68***	.841
2	Thoughts drifting beyond control	*C1*	.834	.020	41.74	.266	.049	5.42***	.767
8	Becoming lost in fantasies	*C1*	.818	.018	45.62	.034	.051	0.67	.670
15	Emotional distance from others	*C2*	.671	.028	23.98	.496	.033	15.16***	.696
14	Limiting interactions to minimum	*C2*	.734	.024	31.14	.449	.030	15.16***	.740
20	Avoiding contact with others	*C2*	.622	.031	20.34	.545	.032	17.05***	.684
10	Becoming cynical; relationships lose meaning	*C2*	.727	.025	28.76	.398	.032	12.26***	.687
9	Preoccupied with own affairs; wanting to be left alone	*C2*	.546	.036	15.11	.599	.035	17.32***	.658
13	Others seem particularly difficult	*C2*	.690	.026	26.14	.354	.035	10.20***	.602

Note. Sp. = specific factor assignment (C1 = Detachment; C2 = Emotional Numbness). All items treated as ordered categorical; WLSMV estimator; polychoric correlation matrix. G = general depersonalization factor; λ = standardized factor loading; SE = standard error; R^2 = proportion of item variance explained by all factors jointly. All factors constrained to be mutually orthogonal (bifactor parameterization). * $p < .05$; ** $p < .01$; *** $p < .001$
